# Overcoming a trial ‘adverse event’: impact and experiences of a temporary suspension of randomisation in clinical trials

**DOI:** 10.1186/1745-6215-16-S2-P178

**Published:** 2015-11-16

**Authors:** Lynda Constable, Tracey Davidson, Samantha Wileman, Kirsty McCormack, Ruth Thomas, Alison McDonald, Cathryn Glazener, John Norrie

**Affiliations:** 1 University of Aberdeen, Aberdeen, UK

## 

A number of factors can result in the temporary suspension of randomisation in clinical trials, with the most common being safety, effectiveness and recruitment. However, external factors such as commercial, political, patient and media pressure can also have a significant impact on decisions to temporarily suspend randomisation.

When the decision has been made to suspend randomisation, ethical considerations and future trial standing need to be addressed. This is not disputed when there is a clear and differential effect that needs to be reviewed. For example, a temporary suspension to evaluate unacceptable risks/undesirable consequences may be more ethical than continuing to recruit. However, imposing a temporary suspension of a trial too early may result in data that cannot be reliably interpreted. It may be more ethical to continue with the trial until this perceived effect is properly evaluated and interpreted. Information given to and the potential effect on all stakeholders (including patients and trial sites) should be considered as well as future recruitment, particularly where the decision is based on limited data.

Results/reasons for a temporary trial suspension are not widely published (if at all), making it difficult to fully interpret any findings. The need to report the results or details of such a temporary suspension is arguably as important as the findings of completed studies.

This presentation will discuss the implications, challenges and lessons learnt in delivering a trial following a temporary suspension of randomisation due to safety concerns.

